# Interactive effect of high sodium intake with increased serum triglycerides on hypertension

**DOI:** 10.1371/journal.pone.0231707

**Published:** 2020-04-16

**Authors:** Jong Wook Choi, Joon-Sung Park, Chang Hwa Lee

**Affiliations:** 1 Research Institute of Medical Science, Konkuk University School of Medicine, Chungju, Korea; 2 Department of Internal Medicine, Hanyang University College of Medicine, Seoul, Korea; Shanghai Institute of Hypertension, CHINA

## Abstract

**Background:**

A high salt diet is associated with the development of hypertension, one of the most important cardiovascular risk factors. A reduction in sodium intake seems to have an effect on increasing serum triglycerides (TGs). Elevated TGs are independently linked to cardiovascular risk. However, there is limited evidence of a possible interactive effect of sodium intake and serum TGs on high blood pressure (BP).

**Methods:**

We conducted a nationwide, population-based interaction analysis using the Kawasaki method for estimating 24-h urinary sodium excretion (e24hUNaE_Kawasaki_) as a candidate indicator of dietary sodium intake. All native Koreans aged 20 years or older without significant medical illness were eligible for inclusion.

**Results:**

A total of 16936 participants were divided into quintiles according to their e24hUNaE_Kawasaki_ results. Participants in the highest quintile were more obese and hypertensive and had higher white blood cell count, lower hemoglobin, greater glycemic exposure, and poor lipid profiles compared to the same parameters of individuals in other quintiles. Linear regression revealed that e24UNaE_Kawasaki_ was related to systolic BP, diastolic BP, and TGs. Multiple logistic regression, adjusted for dietary sodium intake and various conventional risk factors for chronic vascular diseases, showed that both e24UNaE_Kawasaki_ and TGs were significant predictors of hypertension. Our interaction analysis demonstrated that increased sodium intake was associated with higher risk of hypertension in participants with elevated TGs than in those without (adjusted RERI = 0.022, 95% *CI* = 0.017–0.027; adjusted AP = 0.017, 95% *CI* = 0.006–0.028; adjusted SI = 1.010, 95% *CI* = 1.007–1.014).

**Conclusion:**

Our findings suggest that the interaction between a high salt diet and elevated TGs may exert synergistic biological effects on the risk of hypertension.

## Introduction

Hypertension (HTN), one of the most well-known chronic diseases, has a multifactorial nature linked to the possible interaction of genetic and environmental risk factors [1, 2]. Furthermore, HTN is a very important modifiable risk factor for cardiovascular morbidity and mortality [3, 4]. Identifying and controlling various risk factors for HTN is critical to prevent its irreversible consequences and to guide treatment decisions.

Previous studies reported not only that high sodium intake was strongly related to the development of HTN and increased risk of cardiovascular diseases (CVDs) but also that dietary intervention could decrease arterial blood pressure (BP) and reduce left ventricular mass in hypertensive individuals [5–9]. These findings suggest that an excess sodium diet is one of the most important modifiable risk factors for HTN and other CVDs. In this respect, the accurate estimation of dietary sodium intake is critical to reduce future cardiovascular outcomes. While 24-h urine collection is regarded as a proper method for assessing 24-h urinary sodium excretion and dietary sodium intake, it is often unfeasible in clinical practice due to high participant burden [10, 11]. To overcome its drawbacks, several mathematical estimation models, including the Kawasaki, Tanaka, and Mage methods, have been introduced to estimate dietary sodium intake from spot urine samples [12, 13]. Nevertheless, the validity of these estimated 24-h urinary sodium excretion (e24hUNaE) methods in relation to arterial BP remains to be evaluated.

Recent clinical studies have suggested that there is a close relationship between an increased sodium diet and abnormal lipid metabolism in the pathogenesis of CVDs, showing that not only subjects consuming increased dietary sodium were more obese and had a tendency to have a poor lipid profile but also that the coexistence of salt sensitivity and metabolic disturbance contributed to more-severe target organ damage [14–16]. Moreover, hypertriglyceridemia, a component of dyslipidemia contributing to adverse cardiovascular events, has been associated with a reduction in sodium intake [17, 18]. Nevertheless, there is limited clinical evidence of the possible interaction of high sodium intake and hypertriglyceridemia in the development of arterial HTN. We sought to find an optimal equation to estimate 24-h urine sodium excretion and explore the interactive effect of sodium intake and serum TG on elevated BP.

## Subjects and methods

### Study population

All data were collected from public-use data sets from the Korean National Health and Nutrition Examination Survey (KNHANES) conducted by the Korea Centers for Disease Control and Prevention (KCDC) with noninstitutionalized Korean civilians. All the participants were volunteers and provided written informed consent before enrollment in the study. Their records, except for the date of the survey, were anonymized prior to analysis. The KNHANES survey was conducted after reviewing and receiving approval from the Institutional Review Board (IRB) of the Korean Centers for Disease Control and Prevention annually. (IRB: 2009-01CON-03-2C, 2010-02CON-21-C, 2011-02CON-06-C, 2013-12EXP-03-5C). Because this retrospective study used and analyzed only publicly available datasets from the KNHANES survey, further specific ethical approval was exempted from the IRB.

A total of 46749 individuals participated in the KNHANES VI-IV. Individuals were excluded from the present analysis for any of the following reasons: (1) incomplete anthropometric or laboratory data, (2) < 20 years of age, (3) pregnancy, (4) a history of medical problems including diabetes, malignancy, and dyslipidemia (5) estimated glomerular filtration rate (eGFR) < 60 mL/min/1.73 m^2^, or (6) urine albumin/creatinine ratio (UACR) > 30 mg/g creatinine.

Because our receiver operating characteristic (ROC) curve analysis demonstrated that the Kawasaki method for estimating 24-h urinary sodium excretion (e24hUNaE_Kawaski_) had the best precision in estimating the effect of high sodium intake on HTN, the final 16936 participants were divided into quintiles according to their e24hUNaE_Kawaski_ results stratified by sex (S1 Fig and S2 Fig).

### Anthropometric and clinical measurements

Anthropometric measurements were performed by well-trained examiners. Participants wore a lightweight gown or underwear. Height (Ht) was measured to the nearest 0.1 cm using a portable stadiometer (Seriter, Bismarck, ND). Weight was measured to the nearest 0.1 kg on a calibrated balance-beam scale (Giant-150N; Hana, Seoul, Korea). Waist circumference (WC) was measured using a flexible tape at the narrowest point between the lowest border of the rib cage and the uppermost lateral border of the iliac crest at the end of normal expiration. Body mass index (BMI) was calculated as weight in kilograms divided by square of the Ht in meters.

BP was measured three times using a mercury sphygmomanometer (Baumanometer; Baum, Copiague, NY) while subjects were in a sitting position following a 5-minute rest period. The average values of the 3 recorded systolic and diastolic BPs were used in the analysis. The quality control analysis of blood pressure measurement was performed and digit preference scoring was monitored [19].

### Nutrition assessment

Nutrient intake levels, including total calorie (Kcal/day), protein (g/day), fat (g/day), carbohydrate (g/day), and sodium (g/day) intakes, were assessed using semiquantitative 24-h dietary recall methods administered by trained interviewers. The food-frequency questionnaire consisted of the type, amount, and frequency of foods or drinks consumed during the previous day and was conducted via face-to-face interviews. Data on food items were converted to units of nutrients using the Food Composition Table developed by the National Institute of Agricultural Sciences (7th revision) and the database of the Korean Health Industry Development Institute for instant foods and imported foods [20].

### Laboratory tests

Venous blood samples were collected after 8 h of overnight fasting. Fasting plasma concentrations of glucose, TGs, high-density lipoprotein (HDL)-cholesterol, and low-density lipoprotein (LDL) cholesterol were determined by a Hitachi Automatic Analyzer 7600 (Hitachi, Tokyo, Japan). Glycated hemoglobin (HbA1c) levels were determined by high-performance liquid chromatography with an automated HLC-723G7 analyzer (Tosoh Corporation, Tokyo, Japan). Serum creatinine levels were measured colorimetrically (Hitachi Automatic Analyzer 7600), and eGFR was calculated using the Chronic Kidney Disease Epidemiology Collaboration equation [21]. To obtain the UACR, urinary albumin was measured in fasting morning spot urine using the immunoturbidimetric method, and urinary creatinine was measured using the colorimetric method. Urine sodium concentrations were measured using an ion-selective electrode, and 24-h sodium excretion was estimated based on concentrations of sodium and creatinine in fasting morning spot urine specimens according to 3 different methods (S1 Table).

### Definition

According to the European Society of Cardiology and European Society of Hypertension (ESC/ESH) guidelines in 2018 and the Korean Society of Hypertension guidelines in 2018 [22, 23], HTN was defined as either the use of antihypertensive therapy, systolic BP above 140 mmHg or diastolic BP above 90 mmHg.

According to previous Clinical Practice Guidelines [24], hypertriglyceridemia was defined as a serum TG level of 150 mg/dL or more.

### Statistical analysis

All data, including sociodemographic information, medical conditions, anthropometric and clinical measurements, and laboratory results, are presented as the mean ± SE or frequencies (and proportions). The generalized linear model was used to compare quantitative variables, and the chi-square test was used to compare proportions for categorical variables. Linear regression analysis was used to assess the relationship between potential predictor variables of HTN and e24hUNaE_Kawaski_. Restricted cubic spline (RCS) regression analysis was used to identify possible nonlinear dependency of the association between candidate risk factors and an increased risk of impaired kidney function [25].

Odds ratios (ORs) with 95% confidence intervals (*CI*s) were calculated in multiple logistic regression models according to the presence of HTN. Receiver operating characteristic (ROC) curves were used to compare the predictive capacity of 3 different methods to assess e24hUNaE in regard to HTN. Nonparametric methods previously described by DeLong et al were used to compare the area under the ROC curves (AUCs) [26].

The biological interactive effect between candidate risk factors on dependent variables was evaluated on both multiplicative scales and additive scales using logistic regression analysis [27, 28]. The interaction analysis based on a multiplicative scale was performed by comparing participants in the highest e24hUNaE_Kawaski_ or TG quintiles to those in the lower e24hUNaE_Kawaski_ and TG quintiles. The interaction based on the additive scale was evaluated by 3 indices: RERI, the relative excess risk due to the interaction; AP, the attributable proportion due to the interaction; and SI, the additive interaction index of synergy.

To control the unmeasured confounding effect(s) related to antihypertensive medication, we performed propensity score-based sensitivity analysis.

A two-tailed P < 0.05 was considered statistically significant. Statistical Analysis Software version 9.4 (SAS Institute Inc, Cary, NC, USA) was used for all analyses.

## Results

### Baseline characteristics

The participants (n = 16,936) comprised 7,721 men and 9,215 women with a mean age of 44.7 ± 14.9 years and a total of 5,844 participants were taking antihypertensive medication. They were divided into quintiles according to their e24UNaE_Kawasaki_ results and were stratified by sex. Participants in the highest e24UNaE_Kawasaki_ quintile were older and more obese, and they were more likely to have elevated BP, lower hemoglobin, greater glycemic exposure, poor lipid profiles, and higher UACR. Interestingly, subjects who consumed a high sodium diet had a tendency to consume a lower fat and higher carbohydrate diet. The other demographic data and clinical characteristics are presented in Table 1.

**Table 1:** General characteristics grouped according to e24UNaE_Kawasaki_**.

	Quintile 1	Quintile 2	Quintile 3	Quintile 4	Quintile 5	
e24UNaE_Kawasaki_ in male (g/day)	≥ 0.5, ≤ 3.2	> 3.2, ≤ 3.9	> 3.9, ≤ 4.6	> 4.6, ≤ 5.4	> 5.4, ≤ 17.4	
e24UNaE_Kawasaki_ in female (g/day)	≥ 0.6, ≤ 2.8	> 2.8, ≤ 3.5	> 3.5, ≤ 4.1	> 4.1, ≤ 4.9	> 4.9, ≤ 12.7	
Variables	(n = 4093)	(n = 4091)	(n = 4095)	(n = 4095)	(n = 4088)	P
Age (year)	39.2 ± 0.3	41,4 ± 0.3	43.5 ± 0.3	45.0 ± 0.3	46.1 ± 0.3	<0.0001
Sex (% male)	1873 (46)	1871 (46)	1874 (46)	1872 (46)	1871 (46)	0.9365
Current smoker (%)	1235 (30)	1152 (28)	1063 (26)	1039 (25)	1073 (26)	0.0015
Systolic blood pressure (mmHg)	113.2 ± 0.2	115.1 ± 0.2	117.1 ± 0.3	119.0 ± 0.3	122.2 ± 0.3	<0.0001
Diastolic blood pressure (mmHg)	73.7 ± 0.2	74.4 ± 0.2	75.6 ± 0.2	76.3 ± 0.2	77.6 ± 0.2	<0.0001
Body mass index (kg/m^2^)	22.6 ± 0.1	23.1 ± 0.1	23.5 ± 0.1	23.9 ± 0.1	24.4 ± 0.1	<0.0001
Waist circumference (cm)	77.7 ± 0.2	79.1 ± 0.2	80.6 ± 0.2	81.9 ± 0.2	83.5 ± 0.2	<0.0001
White blood cell count (10^9^/L)	6.44 ± 0.03	6.26 ± 0.03	6.29 ± 0.03	6.24 ± 0.03	6.27 ± 0.03	<0.0001
Hemoglobin (g/dL)	14.36 ± 0.03	14.24 ± 0.03	14.21 ± 0.03	14.21 ± 0.03	14.18 ± 0.03	0.0005
Platelets (10^3^/μL)	263.2 ± 1.1	259.3 ± 1.1	258.6 ± 1.0	255.6 ± 1.1	256.6 ± 1.1	<0.0001
eGFR* (mL/min/1.73 m^2^)	101.4 ± 0.3	101.5 ± 0.3	100.5 ± 0.3	100.1 ± 0.3	101.5 ± 0.3	0.0073
Fasting glucose (mg/dL)	96.3 ± 0.4	96.1 ± 0.3	97.3 ± 0.3	97.9 ± 0.3	98.9 ± 0.3	<0.0001
Hemoglobin A1c (%)	5.66 ± 0.02	5.67 ± 0.02	5.72 ± 0.02	5.77 ± 0.02	5.68 ± 0.02	<0.0001
Aspartate aminotransferase (IU/L)	21.9 ± 0.2	21.7 ± 0.2	22.2 ± 0.2	22.7 ± 0.2	23.2 ± 0.2	<0.0001
Alanine aminotransferase (IU/L)	20.9 ± 0.3	20.6 ± 0.2	21.8 ± 0.3	22.8 ± 0.3	23.3 ± 0.3	<0.0001
Triglycerides (mg/dL)	118.9 ± 1.6	124.7 ± 1.7	133.0 ± 1.8	140.1 ± 2.1	149.5 ± 2.1	<0.0001
HDL cholesterol (mg/dL)	51.3 ± 0.2	50.4 ± 0.2	49.7 ± 0.2	49.1 ± 0.2	48.8 ± 0.2	<0.0001
LDL cholesterol (mg/dL)	110.5 ± 0.7	110.9 ± 0.8	120.0 ± 0.8	111.8 ± 0.8	112.1 ± 0.8	0.1047
UACR (mg/g Cr)	13.0 ± 1.9	11.4 ± 1.0	17.5 ± 3.2	19.9 ± 3.8	27.3 ± 4.6	<0.0001
Dietary intake						
Total calories (Kcal/day)	2046 ± 17	2082 ± 17	2095 ± 15	2103 ± 18	2091 ± 17	0.0129
Protein intake (g/day)	71.8 ± 0.7	74.2 ± 0.8	75.3 ± 0.8	75.1 ± 0.9	74.1 ± 0.8	0.0082
Fat intake (g/day)	47.4 ± 0.6	46.9 ± 0.6	46.1 ± 0.6	44.8 ± 0.7	42.6 ± 0.6	<0.0001
Carbohydrate intake (g/day)	310.8 ± 2.2	318.3 ± 2.2	323.5 ± 2.1	329.5 ± 2.3	331.6 ± 2.4	<0.0001
Sodium intake (g/day)	4.19 ± 0.05	4.48 ± 0.05	4.61 ± 0.05	4.86 ± 0.07	4.91 ± 0.06	<0.0001
Potassium intake (g/day)	2.95 ± 0.03	3.04 ± 0.03	3.13 ± 0.03	3.16 ± 0.03	3.12 ± 0.03	<0.0001
Alcohol intake (g/day)	2.2 ± 0.1	2.3 ± 0.1	2.3 ± 0.1	2.4 ± 0.1	2.6 ± 0.1	<0.0001
Morning fasting urine						
Urine sodium (mmol/L)	80.4 ± 05	112.7 ± 0.6	128.9 ± 0.7	144.6 ± 0.8	162.4 ± 1.0	<0.0001
Urine creatinine (mmol/L)	22.3 ± 0.2	16.4 ± 0.1	13.4 ± 0.1	11.0 ± 0.1	7.9 ± 0.1	<0.0001
Urine Na/Cr ratio (mmol/mmol)	3.93 ± 0.02	7.25 ± 0.03	10.23 ± 0.04	14.11 ± 0.06	23.37 ± 0.14	<0.0001
Estimated 24-h urine sodium excretion (e24UNaE)						
e24UNaE_Kawasaki_** (g/day)	2.458 ± 0.007	3.382 ± 0.004	4.006 ± 0.005	4.710 ± 0.006	6.057 ± 0.014	<0.0001
e24UNaE_Takada_*** (g/day)	2.129 ± 0.005	2.751 ± 0.003	3.152 ± 0.003	3.589 ± 0.003	4.370 ± 0.008	<0.0001
e24UNaE_Mage_**** (g/day)	0.794 ± 0.005	1.458 ± 0.004	2.040 ± 0.006	2.819 ± 0.010	4.754 ± 0.029	<0.0001

The results are expressed as the mean ± SD or frequencies (and proportions).

eGFR, estimated glomerular filtration rate; HDL, high-density lipoprotein; LDL, low-density lipoprotein; Na, sodium; Cr, creatinine; UACR, urine albumin/Cr ratio.

*Estimated using the Chronic Kidney Disease Epidemiology Collaboration equation.

** e24UNaE calculated using the Kawasaki method.

*** e24UNaE calculated using the Takada method.

**** e24UNaE calculated using the Mage method.

### Relationship of dietary salt intake with HTN-related risk factors

**Fig 1:**
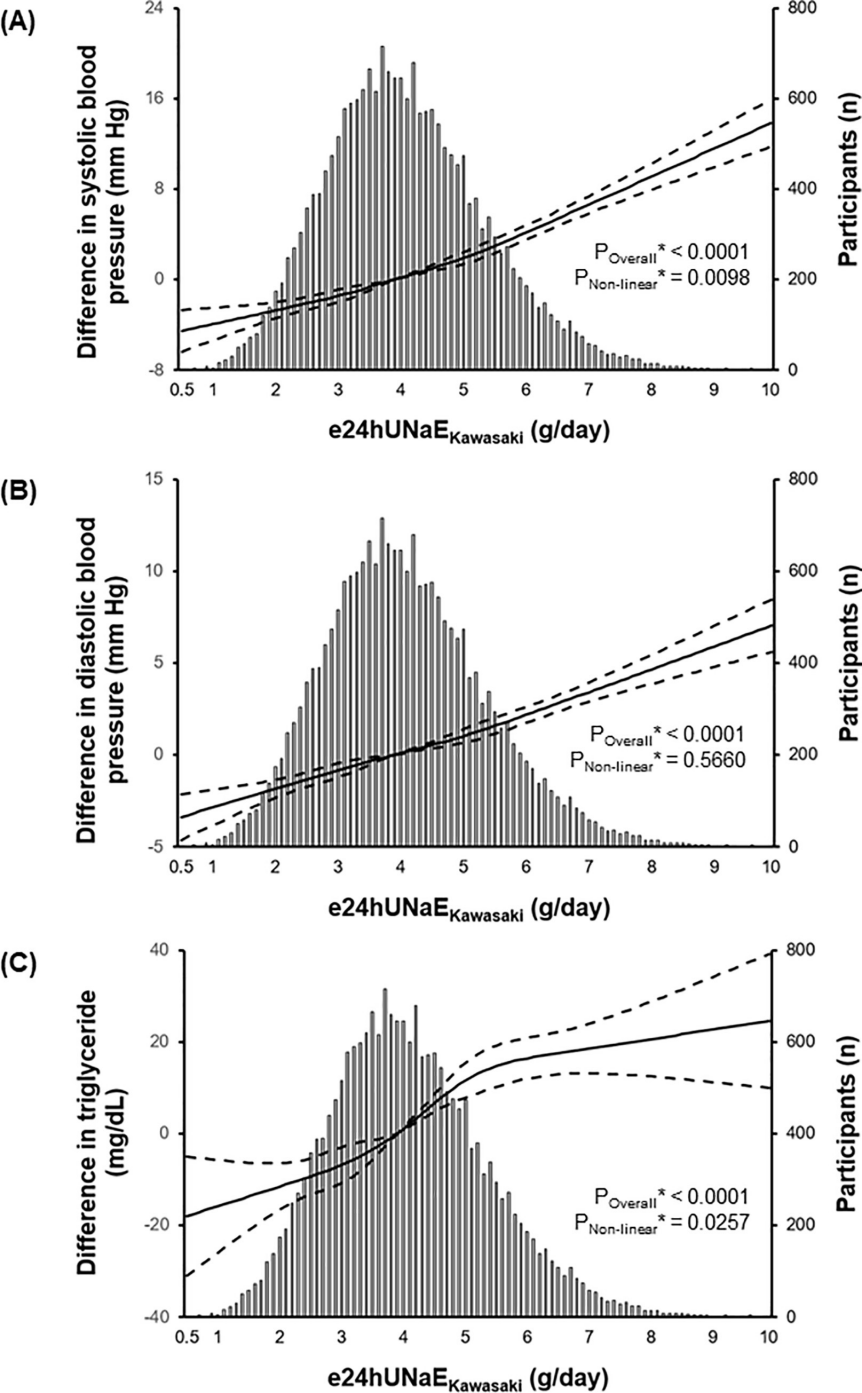
Relationship of e24UNaE_Kawasaki_^#^ with difference in (A) systolic blood pressure, (B) diastolic blood pressure, and (C) serum triglycerides compared with the chosen reference e24UNaE_Kawasaki_ of 3.9. The solid line represents the difference in systolic blood pressure, diastolic blood pressure, and triglycerides, and the dashed lines represent 95% confidential intervals. Histogram illustrates the distribution of e24UNaE_Kawasaki_ among participants. *Calculated by linear regression model of adjusted restricted cubic spline analysis using age, sex, and smoking history as covariates. ^#^Estimated 24-h urinary sodium excretion calculated using the Kawasaki method.

**Fig 2:**
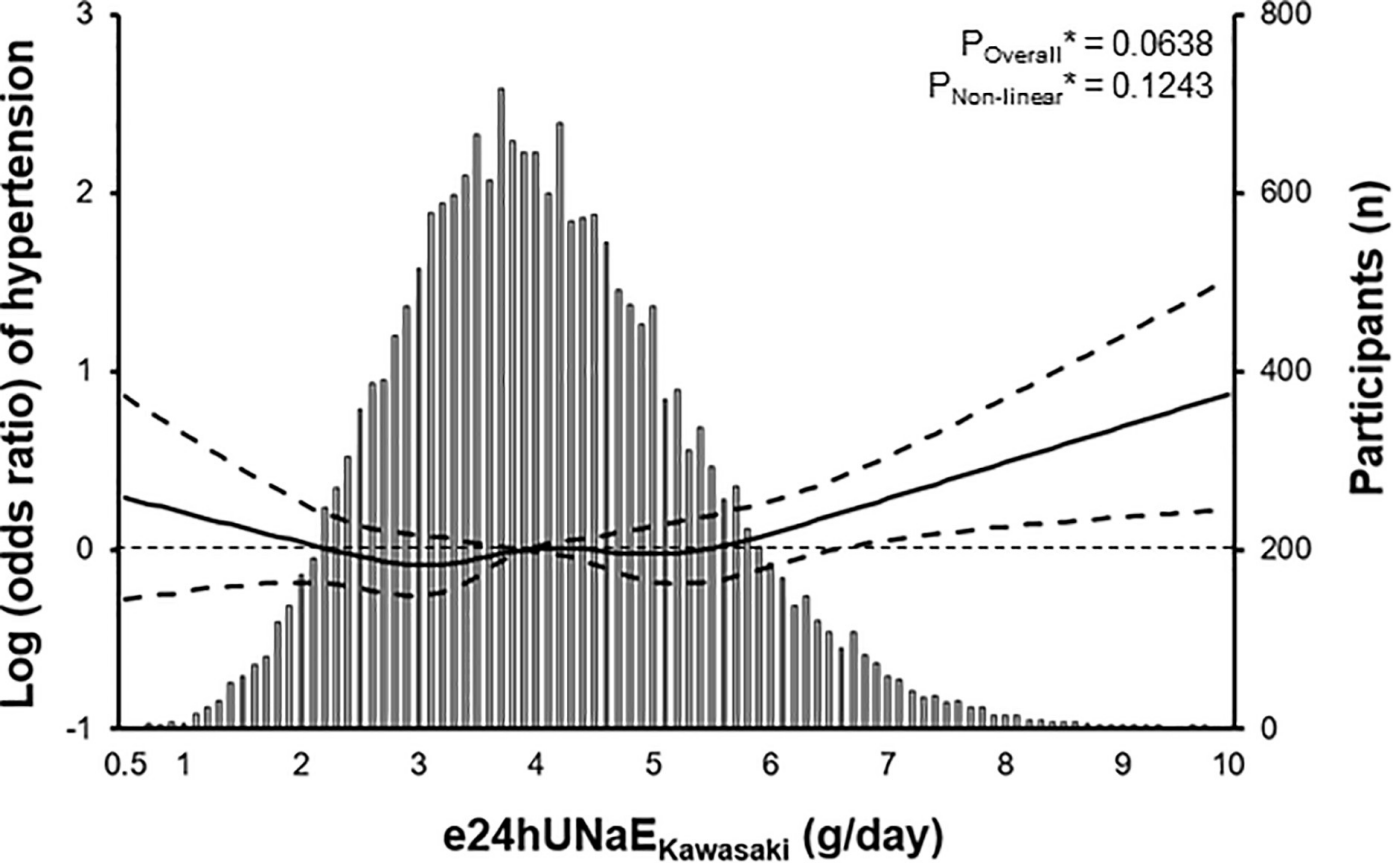
Nonlinear relationship between e24UNaE_Kawasaki_ and the odds ratio of hypertension^#^ with the chosen reference e24UNaE_Kawasaki_ of 3.9. The solid line represents the odds ratio of hypertension, and the dashed lines represent 95% confidential intervals. Histogram illustrates the distribution of e24UNaE_Kawasaki_ among participants. *Calculated by linear regression model of adjusted restricted cubic spline analysis using age, sex, and smoking history as covariates and body mass index, waist circumference, white blood cell count, hemoglobin, fasting plasma glucose, hemoglobin A1c, aspartate aminotransferase, alanine aminotransferase, UACR, and daily alcohol intake as predictors. ^#^Defined as the use of antihypertensive therapy, systolic BP above 140 mmHg or a diastolic BP above 90 mmHg. UACR, urine albumin/Cr ratio.

We performed linear regression and RCS analyses with age, sex, and smoking history as covariates to find the possible relation of e24UNaE_Kawasaki_ with other baseline characteristics related to vascular endothelial function. As shown in S2 Table and Fig 1, we found that e24UNaE_Kawasaki_ was deeply related to dietary nutrient intake and several components of metabolic syndrome, including systolic BP, diastolic BP, and TGs. The relationship between e24UNaE_Kawasaki_ and the odds ratio of hypertension with the chosen reference e24UNaE_Kawasaki_ of 3.9 was shown in Fig 2.

### Association of dietary sodium intake with hypertriglyceridemia and HTN

We performed multiple logistic regression models, using age, sex, and smoking as covariates, to find the exact association of e24UNaE_Kawasaki_ with HTN and related risk factors. First, we found that e24UNaE_Kawasaki_ was an independent risk factor for hypertriglyceridemia (adjusted OR = 1.251, 95% *CI* = 1.215–1.287, S3 Table). Of note, we also found that either e24UNaE_Kawasaki_ or serum TG levels were significantly associated with overt HTN, and further adjustment for BMI, WC, white blood cell count, hemoglobin, eGFR, fasting plasma glucose, hemoglobin A1c, aspartate aminotransferase, alanine aminotransferase, UACR, daily alcohol intake, and dietary sodium intake as predictors did not attenuate these associations (e24UNaE_Kawasaki_, adjusted OR = 1.107, 95% *CI* = 1.027–1.210; TG, adjusted OR = 1.001, 95% *CI* = 1.001–1.002; Table 2). Interestingly, our results demonstrated that the OR of e24UNaE was increased after additional adjustment for TGs in logistic regression analysis, and there was a J-shaped association between dietary sodium intake and the risk of HTN in the RCS analysis, suggesting that a possible interaction between increased sodium intake and elevated TG levels may contribute to a higher risk of HTN.

**Table 2:** Multivariable logistic regression for hypertension*.

	Crude			Model I			Model II		
Variable	OR	95% *CI*	P	OR	95% *CI*	P	OR	95% *CI*	P
Age (year)	1.063	1.060–1.066	<0.0001						
Female (vs. male)	0.624	0.578–0.672	<0.0001						
Smoker (vs. nonsmoker)	1.204	1.106–1.310	<0.0001						
Body mass index (kg/m^2^)	1.180	1.165–1.197	<0.0001						
Waist circumference (cm)	1.063	1.059–1.068	<0.0001						
White blood cell count (10^9^/L)	1.106	1.078–1.134	<0.0001						
Hemoglobin (g/dL)	1.348	1.290–1.409	<0.0001						
Platelets (10^3^/μL)	0.999	0.998–0.999	<0.0001						
eGFR (mL·min^-1^·1.73 m^-2^)	0.954	0.951–0.957	<0.0001						
Fasting plasma glucose (mg/dL)	1.058	1.053–1.063	<0.0001						
Hemoglobin A1c (%)	3.347	2.794–4.009	<0.0001						
Aspartate aminotransferase (IU/L)	1.030	1.024–1.036	<0.0001						
Alanine aminotransferase (IU/L)	1.015	1.012–1.018	<0.0001						
Triglycerides (mg/dL)	1.003	1.003–1.004	<0.0001	1.001	1.001–1.002	<0.0001			
HDL cholesterol (mg/dL)	0.978	0.974–0.982	<0.0001	0.998	0.990–1.007	0.7126			
LDL cholesterol (mg/dL)	1.008	1.006–1.010	<0.0001	1.002	0.996–1.008	0.4896			
UACR (mg/g Cr)	1.079	1.065–1.093	<0.0001						
Dietary intake									
Total calories (Kcal/day)	1.001	1.001–1.001	<0.0001	1.000	0.999–1.001	0.3397			
Protein intake (g/day)	0.998	0.997–0.999	<0.0001	1.000	0.999–1.001	0.4649			
Fat intake (g/day)	0.992	0.990–0.994	<0.0001	1.000	0.998–1.001	0.5359			
Carbohydrate intake (g/day)	1.000	0.999–1.001	0.3327						
Sodium intake (g/day)	1.001	0.990–1.013	0.8377						
Potassium intake (g/day)	0.973	0.948–0.999	<0.0001	0.983	0.954–1.012	0.2398			
Alcohol intake (g/day)	1.096	1.083–1.108	<0.0001	1.091	1.076–1.107	<0.0001			
Estimated 24-h urine sodium excretion									
e24UNaE_Kawasaki_ (g/day)	1.241	1.203–1.281	<0.0001	1.107	1.024–1.197	0.0107	1.115	1.027–1.210	0.0092
e24UNaE_Tanaka_ (g/day)	1.454	1.382–1.530	<0.0001	1.142	1.008–1.295	0.0376	1.157	1.015–1.320	0.0294
e24UNaE_Mage_ (g/day)	1.148	1.116–1.181	<0.0001	1.095	1.028–1.166	0.0050	1.104	1.033–1.181	0.0039

* Defined as either the use of antihypertensive therapy and/or systolic BP above 140 mm Hg or a diastolic BP above 90 mm Hg.

Model I, performed using age, sex, and smoking as covariates and body mass index, waist circumference, white blood cell count, hemoglobin, eGFR, fasting plasma glucose, hemoglobin A1c, aspartate aminotransferase, alanine aminotransferase, UACR, and daily alcohol intake as predictors.

Model II, performed using age, sex, and smoking as covariates and body mass index, waist circumference, white blood cell count, hemoglobin, eGFR, fasting plasma glucose, hemoglobin A1c, aspartate aminotransferase, alanine aminotransferase, triglycerides, UACR, and daily alcohol intake as predictors.

OR, odds ratio; *CI*, confidence interval.

### Biological interaction analysis of the interactive effect of high sodium intake and increased triglyceride level on HTN

**Fig 3:**
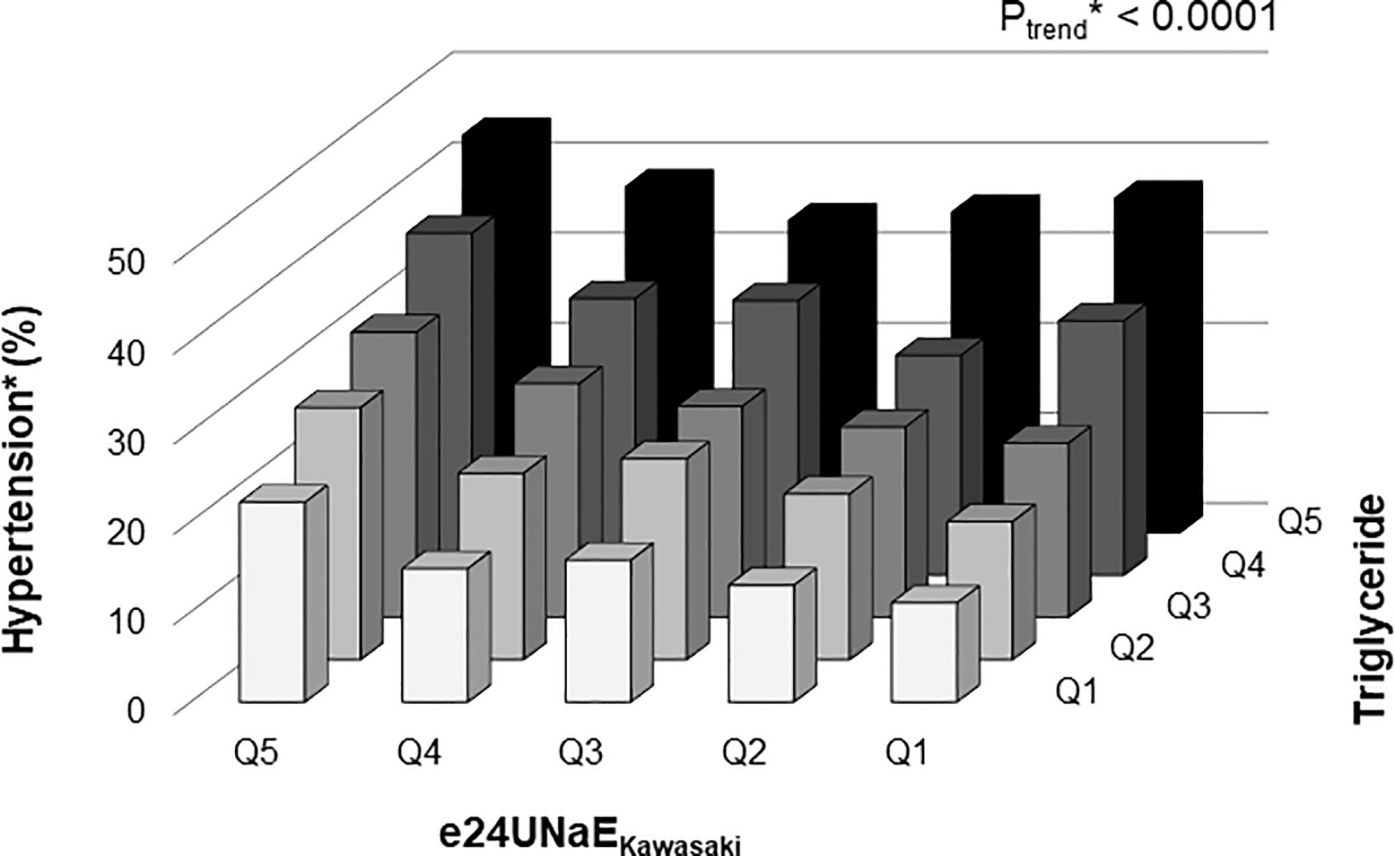
Prevalence of hypertension^#^ according to e24UNaE_Kawasaki_ and serum triglyceride quintile. *Estimated using the Cochran**-**Armitage test for trend.

To explore the interactive effects of high sodium intake and elevated TG levels on the development of HTN, all participants were further classified into TG quintiles according to their TG levels, and we conducted the Cochran**-**Armitage test for trends and multiplicative and additive interaction analyses. As shown in Fig 3 and Table 3, there was a trend toward an increasing prevalence of HTN with increasing e24UNaE_Kawasaki_ and TG quintiles, and the relative risk of HTN was higher in participants in both the highest quintiles (crude OR = 3.331, 95% *CI* = 2.976–3.727). Of note, this multiplicative interaction remained after further adjustment for age, sex, smoking, BMI, WC, white blood cell count, hemoglobin, eGFR, fasting plasma glucose, hemoglobin A1c, aspartate aminotransferase, alanine aminotransferase, UACR, daily alcohol intake, and dietary sodium intake (adjusted OR = 1.850, 95% *CI* = 1.018–3.361).

**Table 3:** Interactive effect analysis of high sodium intake and increased triglyceride level on hypertension.

Categories		Unadjusted			Adjusted*		
Triglyceride quintile	e24UNaE_Kawasaki_ quintile	OR	95% *CI*	P	OR	95% *CI*	P
1–3	1–2	1 (reference)			1 (reference)		
1–3	3–5	1.590	1.413–1.790	<0.0001	1.128	0.867–1.467	0.3685
4–5	1–2	2.488	2.183–2.835	<0.0001	1.314	0.972–1.777	0.0762
4–5	3–5	3.331	2.976–3.727	<0.0001	1.526	1.091–2.134	0.0137

*Adjusted for age, sex, smoking history, body mass index, waist circumference, white blood cell count, hemoglobin, eGFR, fasting plasma glucose, hemoglobin A1c, aspartate aminotransferase, alanine aminotransferase, UACR, and daily alcohol intake.

In the biologic interaction analysis (Table 4), we also found that there was a higher risk of HTN in participants with both high sodium intake and elevated serum TG attributed to the synergistic interaction between them, even after adjustment for age, sex, smoking, BMI, WC, white blood cell count, hemoglobin, eGFR, fasting plasma glucose, hemoglobin A1c, aspartate aminotransferase, alanine aminotransferase, UACR, daily alcohol intake, and dietary sodium intake (adjusted RERI = 0.022, 95% *CI* = 0.017–0.027; adjusted AP = 0.017, 95% *CI* = 0.006–0.028; adjusted SI = 1.010, 95% *CI* = 1.007–1.014). In sensitivity analysis conducted between participants between without antihypertensive medication(s) and whole population (S4–S6 Tables), there was no significant difference in both association and interaction of high sodium intake and increased triglyceride level with HTN (P_Sensitivity analysis.Association.e24UNaEKawasak_ = 0.5864, P_Sensitivity analysis.Association.triglyceride_ = 0.6477, P_Sensitivity analysis. Interaction_ = 0.6517).

**Table 4:** Index of additive biological interactive effect of high sodium intake and increased triglyceride level on hypertension.

	Unadjusted		Adjusted*	
Measure	Estimate	95% *CI*	Estimate	95% *CI*
RERI	0.051	0.045–0.056	0.008	0.002–0.014
AP	0.038	0.017–0.058	0.015	0.008–0.022
SI	1.168	1.052–1.284	1.119	1.101–1.137

*Adjusted for age, sex, smoking history, body mass index, waist circumference, white blood cell count, hemoglobin, fasting plasma glucose, hemoglobin A1c, aspartate aminotransferase, alanine aminotransferase, UACR, and daily alcohol intake.

If there was no biological interaction, the 95% *CI* of RERI and AP included 0, and the 95% *CI* of SI contained 1.

RERI, the relative excess risk because of the interaction; AP, the attributable proportion of the interaction; SI, the additive interaction index of synergy.

## Discussion

This study provides comprehensive information on the performance of e24UNaE_Kawasaki_ as a surrogate indicator of dietary sodium intake with respect to HTN. Our results demonstrate not only that e24UNaE_Kawasaki_ was strongly related to both systolic and diastolic BPs but also that the synergistic interaction of a high sodium diet and hypertriglyceridemia was connected with higher risk of HTN.

Adequate sodium intake is critical for BP homeostasis because sodium is an essential component for the regulation of body fluid and osmotic balance and the maintenance of the physiological function of the nervous and muscular systems [29, 30]. Otherwise, excess sodium intake promotes water retention and contributes to salt-sensitive HTN [31]. Another essential effect of sodium on BP may be pro-inflammatory signaling pathway activation and vascular endothelial dysfunction [32–34]. Because such pathologic reactions may lead to the initiation of various chronic illnesses and increased CVD morbidity and mortality [6], it is valuable to find a practical approach to estimate dietary sodium intake for the prediction of adverse health outcomes. However, estimation of the real sodium intake may be inaccurate due to dietary recall bias, errors in self-reporting, or missing data. A significant difference in the sodium levels based on formulas adopted for assessment of dietary sodium intake by fasting morning urinary excretion and dietary intake of sodium based on a questionnaire is probably for this reason, especially when sodium intake was very low or very high in the present study. For the same reason, unlike previous studies, dietary sodium intake was independent of the presence of hypertension in our study. Instead, our linear regression and ROC curve analysis results revealed that e24hUNaE_Kawasaki_ not only had the best precision in estimating the effect of high sodium intake on BP elevation compared to the precision of other methods but could also be of value in indicating its inflammatory effects in the initiation of the systemic vascular response.

In this study, we demonstrated a significant association between high sodium intake and hypertriglyceridemia. There is still controversy regarding the association of dietary sodium intake with lipid dysmetabolism. Previous experimental and epidemiological studies have revealed that the secretion rate of TG was significantly greater in a salt-sensitive animal model, the risk of hypertriglyceridemia was increased in human subjects consuming a high sodium diet, and there was a positive correlation between urinary sodium excretion and TGs and BP [35–37]. On the other hand, recent epidemiologic studies showed that lifestyle modification with reduced dietary salt intake for 4 or more weeks failed to make significant changes in lipid profiles [38]. A previous systematic review showed that sodium reduction increased cholesterols and triglycerides with moderate quality of evidence [18]. Nevertheless, it cannot be overlooked that the meta-analysis was based on randomized controlled studies that mainly targeted the association between low salt diet and blood pressure and only a few studies of Asians. Because all subjects taking lipid-lowering drugs were excluded from our study design and all study subjects were Korean, our results may differ from previous meta-analyses. Further study of the basic mechanisms underlying the potential effects of dietary sodium on lipid metabolism may provide precise insights into the pathogenesis of vascular endothelial dysfunction in subjects consuming a high sodium diet.

Our Cochran-Armitage test and interaction analyses revealed that the biological interaction between high sodium intake and increased TG levels was associated with a higher risk of HTN. Previous experimental and clinical studies have shown that not only direct adverse effects of sodium but also BP-independent biologic mechanisms of sodium, such as decreased bioavailability of vascular nitric oxide, increased generation of reactive oxygen species, and activation of pathologic pro-inflammatory signaling pathways were considerably related to vascular endothelial dysfunction and HTN [39–41]. Oh, et al. recently demonstrated that urinary sodium excretion had a significant association with BP, hypertriglyceridemia, and albuminuria, while these associations changed according to age group [42]. Thus, it can be postulated that changes in dietary habits may modulate the vascular effects associated with abnormal lipid metabolism. However, little is known regarding what basic mechanism related to such pathologic interactions may influence the pathogenesis of vascular endothelial dysfunction.

The relationship between TGs and HTN has been inconsistent in previous studies. Whether high TGs themselves increase BP or are an indicator of HTN is uncertain to date. However, the interaction between TGs and other underlying factors, such as uric acid, glucose, and other cholesterols, seems to elevate BP based on several studies [43–45]. Additionally, Gilbert et al. suggested that a TG-lowering agent reduced BP and renal vasoconstriction only in salt-sensitive subjects, which may suggest an interaction between TG and salt in the context of increased BP and support the validity of our findings [46]. Taken together, these results indicate that high TGs may elevate BP, especially under certain circumstances.

The possible association of e24UNaE_Kawasaki_ with a mild decrease in kidney function, namely, eGFR 60 and 89 mL/min/1.73 m^2^, was not observed in this study. This finding is inconsistent with previous reports showing that there was a weak relationship between urine sodium excretion and kidney dysfunction [47–49]. A possible explanation for this inconsistency is that such a result may be due to the limitation of this cross-sectional study design that cannot efficiently demonstrate the long-term harmful effects of excess sodium intake on the reduction in kidney function. Genetic variation associated with sodium sensitivity of the vascular endothelial system may be another possible explanation of differences in target organ manifestation of a high sodium diet [50].

There were several limitations to our study. First, because this population-based study did not contain data from ambulatory BP monitoring, serum sodium, spot urine potassium, or 24-h urine collection, we could not assess the exact association of poor dietary practices with the salt sensitivity of the vascular endothelium. This limitation made it impossible to investigate the relationship between dietary sodium intake and HTN. Second, the prevalence and severity of HTN are influenced by a wide variety of factors. Because of the limitations of the study design, we could not adjust for many other factors other than age, sex, smoking, BMI, WC, white blood cell count, hemoglobin, eGFR, fasting plasma glucose, hemoglobin A1c, aspartate aminotransferase, alanine aminotransferase, UACR, daily alcohol intake, and dietary sodium intake. Third, because of the self-reported nature of medical history, medication, and use of tobacco and alcohol, a social desirability bias cannot be excluded. Participants may have also forgotten relevant details. This may be responsible for the results and conclusions that conflicted with previous research. Finally, this study was limited by the use of a cross-sectional design, which prevented us from determining cause and effect relationships between salt, TGs, and BP.

The results of our study indicate that high sodium intake is strongly associated with lipid dysmetabolism and that their biological interaction is associated with overt HTN. A large population-based prospective study is warranted to confirm these findings.

## Supporting information

S1 Fig Flow chart of the study group enrollment process. KNHANES, The Korean National Health and Nutritional Examination Survey; Q, estimated 24-h urinary sodium excretion (e24UNaE_Kawasaki_*) quintile. *Estimated 24-h urinary sodium excretion calculated using the Kawasaki method.
(TIF)

S2 Fig Receiver Operating Characteristic (ROC) curves representing the prediction capacity for the risk of hypertension^#^. Compared with other urinary indices for sodium excretion (e24UNaE_Tanaka_, AUC* = 0.5728, 95% CI* = 0.5636–0.5820; e24UNaE_Mage_, AUC* = 0.5642, 95% CI* = 0.5549–0.5734; urinary Na/Cr ratio, AUC* = 0.5524, 95% CI* = 0.5430–0.5617), e24UNaE_Kawasaki_ had the best precision in predicting hypertension (AUC* = 0.5837, 95% CI* = 0.5745–0.5929, P** < 0.0001). *Calculated by logistic regression analysis using age, sex, and smoking history as covariates and body mass index, waist circumference, white blood cell count, hemoglobin, fasting plasma glucose, hemoglobin A1c, aspartate aminotransferase, alanine aminotransferase, UACR, and daily alcohol intake as predictors. **Estimated by nonparametric methods previously described by DeLong et al. ^#^Defined as the use of antihypertensive therapy, systolic BP above 140 mmHg or a diastolic BP above 90 mmHg. e24UNaE_Kawasaki_, estimated 24-h urinary sodium excretion calculated using the Kawasaki method; e24UNaE_Takada_, estimated 24-h urinary sodium excretion calculated using the Takada method; e24UNaE_Mage_, estimated 24-h urinary sodium excretion calculated using the Mage method; Urine Na/Cr ratio, urine sodium/creatinine ratio; AUC, areas under the ROC curves; *CI*, confidence interval; Na, sodium; Cr, creatinine; UACR, urine albumin/Cr ratio.
(TIF)

S1 Table Three methods for estimated 24-h urine sodium excretion (e24UNaE, mg/day). (DOCX)

S2 Table Linear regression for e24UNaE_Kawasaki_ (g/day). (DOCX)

S3 Table Multivariable logistic regression for hypertriglyceridemia. (DOCX)

S4 Table Sensitivity analysis. Multivariable logistic regression for hypertension.
(DOCX)

S5 Table Sensitivity analysis. Interactive effect analysis of high sodium intake and increased triglyceride level on hypertension.
(DOCX)

S6 Table Sensitivity analysis. Index of additive biological interactive effect of high sodium intake and increased triglyceride level on hypertension.
(DOCX)

## Abbreviations and symbols

e24hUNaE: estimated 24-h urinary sodium excretion
KNHANES: Korean National Health and Nutrition Examination Survey
KCDC: Korea Centers for Disease Control and Prevention
eGFR: estimated glomerular filtration rate
UACR: urine albumin/creatinine ratio
CKD-EPI: Chronic Kidney Disease Epidemiology Collaboration equation
RERI: relative excess risk due to the interaction
P: attributable proportion due to the interaction
and SI: additive interaction index of synergy
