# Supplementary figures and images for: Interactive effect of high sodium intake with increased serum triglycerides on hypertension
Source: PLoS One. 2020 Apr 16;15(4):e0231707. doi: 10.1371/journal.pone.0231707 (PMC7162459; doi:10.1371/journal.pone.0231707)

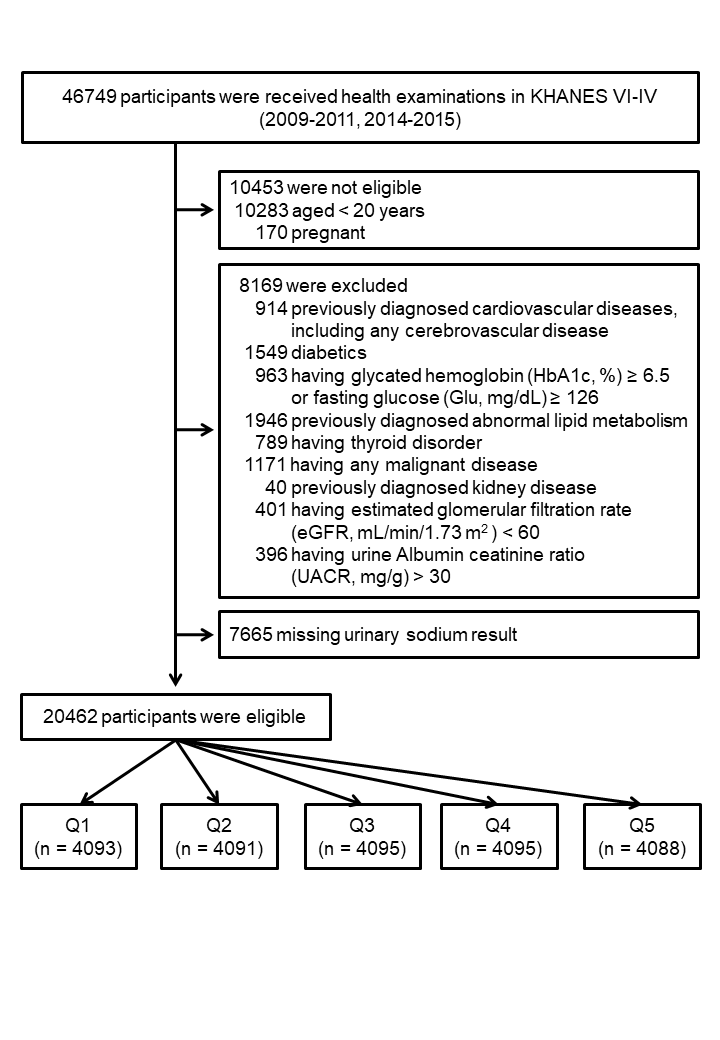

Supplement: S1 Fig — KNHANES, The Korean National Health and Nutritional Examination Survey; Q, estimated 24-h urinary sodium excretion (e24UNaEKawasaki*) quintile. *Estimated 24-h urinary sodium excretion calculated using the Kawasaki method. (TIF) [file pone.0231707.s001.TIF]

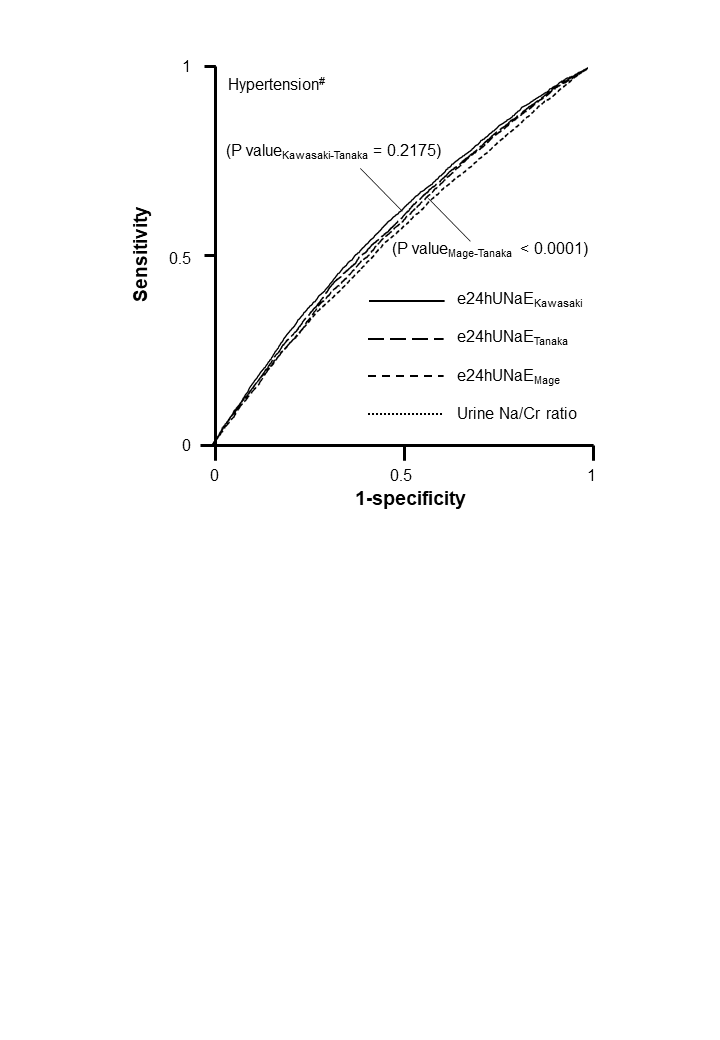

Supplement: S2 Fig — Compared with other urinary indices for sodium excretion (e24UNaETanaka, AUC* = 0.5728, 95% CI* = 0.5636–0.5820; e24UNaEMage, AUC* = 0.5642, 95% CI* = 0.5549–0.5734; urinary Na/Cr ratio, AUC* = 0.5524, 95% CI* = 0.5430–0.5617), e24UNaEKawasaki had the best precision in predicting hypertension (AUC* = 0.5837, 95% CI* = 0.5745–0.5929, P** < 0.0001). *Calculated by logistic regression analysis using age, sex, and smoking history as covariates and body mass index, waist circumference, white blood cell count, hemoglobin, fasting plasma glucose, hemoglobin A1c, aspartate aminotransferase, alanine aminotransferase, UACR, and daily alcohol intake as predictors. **Estimated by nonparametric methods previously described by DeLong et al. #Defined as the use of antihypertensive therapy, systolic BP above 140 mmHg or a diastolic BP above 90 mmHg. e24UNaEKawasaki, estimated 24-h urinary sodium excretion calculated using the Kawasaki method; e24UNaETakada, estimated 24-h urinary sodium excretion calculated using the Takada method; e24UNaEMage, estimated 24-h urinary sodium excretion calculated using the Mage method; Urine Na/Cr ratio, urine sodium/creatinine ratio; AUC, areas under the ROC curves; CI, confidence interval; Na, sodium; Cr, creatinine; UACR, urine albumin/Cr ratio. (TIF) [file pone.0231707.s002.TIF]
