# Supplementary material for: Interactive effect of high sodium intake with increased serum triglycerides on hypertension
Source: PLoS One. 2020 Apr 16;15(4):e0231707. doi: 10.1371/journal.pone.0231707 (PMC7162459; doi:10.1371/journal.pone.0231707)
Supplement: S1 Table — (DOCX) [file pone.0231707.s003.docx]

S1 Table. Three methods for estimated 24-h urine sodium excretion (e24UNaE, mg/day)

| Method | Formula to estimate 24-h urinary sodium excretion | Reference |
| --- | --- | --- |
| Kawasaki | 23 x 16.3 x (Na_spot_ / Cr_spot_ x PrUCr24h)^0.5^ | [10-12] |
|  | PrUCr24h for male = 15.12 x Wt + 7.39 x Ht - 12.63 x age (y) - 79.9 |  |
|  | PrUCr24h for female = 8.58 x Wt + 5.09 x Ht - 4.72 x age (y) - 79.95 |  |
| Tanaka | 23 x 21.98 x (Na_spot_ / Cr_spot_ x PrUCr24h)^0.392^ | [10,12] |
|  | PrUCr24h = 14.89 x Wt + 16.14 x Ht - 2.04 x age (y) - 2244.45 |  |
| Mage | 23 x (Na_spot_ / Cr_spot_ x PrUCr24h) | [11] |
|  | PrUCr24h for male = 0.00179 x [140 - age (y)] x [Wt^1.5^ x Ht^0.5^] x [1 + 0.18 x A* x (1.366 - 0.0159 x BMI)] |  |
|  | PrUCr24h for female = 0.00163 x [140 - age (y)] x [Wt^1.5^ x Ht^0.5^] x [1 + 0.18 x A* x (1.429 - 0.0198 x BMI)] |  |
| Urine Na/Cr ratio | Na_spot_ / Cr_spot_ |  |

*A of African-American of black is 1 and other = 0.

Na_spot_, spot urinary sodium (mmol/L); Cr_spot_, spot urinary creatinine (mmol/L); Wt, weight (kg); Ht, height (cm); PrUCr24h, predicted 24-h urinary creatinine (mg/day); BMI, body mass index (kg/m^2^).
