# Supplementary material for: Interactive effect of high sodium intake with increased serum triglycerides on hypertension
Source: PLoS One. 2020 Apr 16;15(4):e0231707. doi: 10.1371/journal.pone.0231707 (PMC7162459; doi:10.1371/journal.pone.0231707)
Supplement: S2 Table — (DOCX) [file pone.0231707.s004.docx]

S2 Table. Linear regression for e24UNaE_Kawasaki_ (g/day)

|  | Crude | |  | Model I | | |
| --- | --- | --- | --- | --- | --- | --- |
| Variable | Slope | P |  | | Slope | P |
| Age (year) | 0.0109 | <0.0001 |  | |  |  |
| Female (vs. male) | -0.5351 | <0.0001 |  | |  |  |
| Smoker (vs. nonsmoker) | 0.2030 | <0.0001 |  | |  |  |
| Systolic blood pressure (mmHg) | 0.0189 | <0.0001 |  | | 0.0136 | <0.0001 |
| Diastolic blood pressure (mmHg) | 0.0238 | <0.0001 |  | | 0.0152 | <0.0001 |
| Body mass index (kg/m^2^) | 0.0803 | <0.0001 |  | | 0.0642 | <0.0001 |
| Waist circumference (cm) | 0.0346 | <0.0001 |  | | 0.0253 | <0.0001 |
| White blood cell count (10^9^/L) | 0.0048 | 0.4854 |  | |  |  |
| Hemoglobin (g/dL) | -0.0839 | <0.0001 |  | | -0.0694 | <0.0001 |
| Platelets (10^3^/μL) | -0.0015 | <0.0001 |  | | -0.004 | 0.0778 |
| eGFR* (mL/min/1.73 m^2^) | -0.0002 | 0.8425 |  | |  |  |
| Fasting glucose (mg/dL) | 0.0162 | <0.0001 |  | | 0.0065 | <0.0001 |
| Hemoglobin A1c (%) | 0.3305 | <0.0001 |  | | 0.1049 | 0.0271 |
| Aspartate aminotransferase (IU/L) | 0.0091 | <0.0001 |  | | 0.0023 | <0.0001 |
| Alanine aminotransferase (IU/L) | 0.0068 | <0.0001 |  | | 0.0029 | <0.0001 |
| Triglycerides (mg/dL) | 0.0017 | <0.0001 |  | | 0.0010 | <0.0001 |
| HDL cholesterol (mg/dL) | -0.0137 | <0.0001 |  | | -0.0064 | <0.0001 |
| LDL cholesterol (mg/dL) | 0.0009 | 0.1032 |  | |  |  |
| UACR (mg/g Cr) | 0.0211 | <0.0001 |  | | 0.0197 | <0.0001 |
| Dietary intake | | | | | | |
| Total calories (Kcal/day) | 0.0001 | <0.0001 |  | | 0.0001 | 0.0003 |
| Protein intake (g/day) | 0.0021 | <0.0001 |  | | 0.0012 | <0.0001 |
| Fat intake (g/day) | -0.0001 | 0.771 |  | |  |  |
| Carbohydrate intake (g/day) | 0.0011 | <0.0001 |  | | 0.0005 | <0.0001 |
| Sodium intake (g/day) | 0.0525 | <0.0001 |  | | 0.0383 | <0.0001 |
| Potassium intake (g/day) | 0.0604 | <0.0001 |  | | 0.0272 | 0.0002 |
| Alcohol intake (g/day) | 0.0465 | <0.0001 |  | | 0.0208 | <0.0001 |

Model I, adjusted for age, sex, and smoking history.

eGFR, estimated glomerular filtration rate; HDL, high-density lipoprotein; LDL, low-density lipoprotein; UACR, urine albumin/Cr ratio
