# Supplementary material for: Interactive effect of high sodium intake with increased serum triglycerides on hypertension
Source: PLoS One. 2020 Apr 16;15(4):e0231707. doi: 10.1371/journal.pone.0231707 (PMC7162459; doi:10.1371/journal.pone.0231707)
Supplement: S3 Table — (DOCX) [file pone.0231707.s005.docx]

S3 Table. Multivariable logistic regression for hypertriglyceridemia*

|  | Crude | | |  | Model I | | |  | Model IV | | |
| --- | --- | --- | --- | --- | --- | --- | --- | --- | --- | --- | --- |
| Variable | OR | 95% *CI* | P |  | OR | 95% *CI* | P |  | OR | 95% *CI* | P |
| Age (year) | 1.021 | 1.018-1.023 | <0.0001 |  |  |  |  |  |  |  |  |
| Female (vs. male) | 0.335 | 0.310-0.362 | <0.0001 |  |  |  |  |  |  |  |  |
| Smoker (vs. nonsmoker) | 2.382 | 2.197-2.583 | <0.0001 |  |  |  |  |  |  |  |  |
| Systolic blood pressure (mmHg) | 1.034 | 1.031-1.036 | <0.0001 |  | 1.024 | 1.021-1.027 | <0.0001 |  | 1.012 | 0.999-1.025 | 0.0546 |
| Diastolic blood pressure (mmHg) | 1.060 | 1.056-1.064 | <0.0001 |  | 1.046 | 1.044-1.053 | <0.0001 |  | 1.036 | 1.015-1.057 | 0.0007 |
| Body mass index (kg/m^2^) | 1.224 | 1.218-1.255 | <0.0001 |  | 1.210 | 1.194-1.227 | <0.0001 |  |  |  |  |
| Waist circumference (cm) | 1.094 | 1.089-1.100 | <0.0001 |  | 1.080 | 1.075-1.086 | <0.0001 |  |  |  |  |
| White blood cell count (10^9^/L) | 1.318 | 1.286-1.350 | <0.0001 |  | 1.259 | 1.227-1.291 | <0.0001 |  |  |  |  |
| Hemoglobin (g/dL) | 1.533 | 1.492-1.576 | <0.0001 |  | 1.536 | 1.472-1.602 | <0.0001 |  |  |  |  |
| Platelets (10^3^/μL) | 1.004 | 1.003-1.005 | <0.0001 |  | 1.002 | 1.001-1.003 | <0.0001 |  |  |  |  |
| eGFR (mL·min^-1^·1.73 m^-2^) | 0.978 | 0.976-0.981 | <0.0001 |  | 0.997 | 0.993-1.001 | 0.0929 |  |  |  |  |
| Fasting plasma glucose (mg/dL) | 1.057 | 1.052-1.061 | <0.0001 |  | 1.044 | 1.039-1.048 | <0.0001 |  |  |  |  |
| Hemoglobin A1c (%) | 3.762 | 3.217-4.400 | <0.0001 |  | 2.876 | 2.405-3.439 | <0.0001 |  |  |  |  |
| Aspartate aminotransferase (IU/L) | 1.047 | 1.039-1.055 | <0.0001 |  | 1.030 | 1.023-1.037 | <0.0001 |  |  |  |  |
| Alanine aminotransferase (IU/L) | 1.040 | 1.036-1.045 | <0.0001 |  | 1.032 | 1.028-1.037 | <0.0001 |  |  |  |  |
| HDL cholesterol (mg/dL) | 0.910 | 0.905-0.914 | <0.0001 |  | 0.919 | 0.915-0.924 | <0.0001 |  |  |  |  |
| LDL cholesterol (mg/dL) | 1.010 | 1.009-1.012 | <0.0001 |  | 1.008 | 1.006-1.010 | <0.0001 |  |  |  |  |
| UACR (mg/g Cr) | 1.101 | 1.050-1.154 | <0.0001 |  | 1.028 | 1.015-1.042 | <0.0001 |  |  |  |  |
| Dietary intake | | | | | | | | | | | |
| Total calories (Kcal/day) | 1.001 | 1.001-1.001 | <0.0001 |  | 1.000 | 0.999-1.001 | 0.1366 |  |  |  |  |
| Protein intake (g/day) | 1.003 | 1.002-1.004 | <0.0001 |  | 1.000 | 0.999-1.001 | 0.5584 |  |  |  |  |
| Fat intake (g/day) | 1.000 | 0.999-1.001 | 0.8764 |  |  |  |  |  |  |  |  |
| Carbohydrate intake (g/day) | 1.001 | 1.001-1.002 | <0.0001 |  | 1.000 | 0.999-1.001 | 0.6879 |  |  |  |  |
| Sodium intake (g/day) | 1.050 | 1.034-1.066 | <0.0001 |  | 1.011 | 0.997-1.024 | 0.1248 |  |  |  |  |
| Potassium intake (g/day) | 1.073 | 1.049-1.098 | <0.0001 |  | 1.011 | 0.986-1.037 | 0.4011 |  |  |  |  |
| Alcohol intake (g/day) | 1.144 | 1.130-1.157 | <0.0001 |  | 1.087 | 1.073-1.101 | <0.0001 |  |  |  |  |
| Estimated 24-h urine sodium excretion | | | | | | | | | | | |
| e24UNaE_Kawasaki_ (g/day) | 1.266 | 1.078-1.487 | <0.0001 |  | 1.254 | 1.084-1.452 | <0.0001 |  | 1.240 | 1.066-1.443 | 0.0053 |
| e24UNaE_Tanaka_ (g/day) | 1.475 | 1.142-1.905 | <0.0001 |  | 1.455 | 1.151-1.840 | <0.0001 |  | 1.426 | 1.117-1.820 | 0.0044 |
| e24UNaE_Mage_ (g/day) | 1.173 | 1.143-1.205 | <0.0001 |  | 1.164 | 1.024-1.323 | <0.0001 |  | 1.154 | 1.010-1.318 | 0.0350 |

*Defined as a serum triglyceride level of 150 mg/dL or more.

Model I, performed using age, sex, and smoking as covariates

Model IV, performed using age, sex, and smoking as covariates and body mass index, waist circumference, white blood cell, hemoglobin, platelet, fasting plasma glucose, hemoglobin A1c, aspartate aminotransferase, alanine aminotransferase, HDL-cholesterol, LDL-cholesterol, UACR, and daily alcohol intake as predictors.

OR, odds ratio; *CI*, confidence interval.
