# Supplementary material for: Interactive effect of high sodium intake with increased serum triglycerides on hypertension
Source: PLoS One. 2020 Apr 16;15(4):e0231707. doi: 10.1371/journal.pone.0231707 (PMC7162459; doi:10.1371/journal.pone.0231707)
Supplement: S4 Table — Multivariable logistic regression for hypertension. (DOCX) [file pone.0231707.s006.docx]

S4 Table. Sensitivity analysis. Multivariable logistic regression for hypertension*

|  | Crude | |  |  | Model I | |  |  | Model II | |  |
| --- | --- | --- | --- | --- | --- | --- | --- | --- | --- | --- | --- |
| Variable | OR | 95% *CI* | P |  | OR | 95% *CI* | P |  | OR | 95% *CI* | P |
| Participants without antihypertensive medication | | | | | | | | | | | |
| Triglyceride (mg/dL) | 1.003 | 1.003-1.004 | <0.0001 |  | 1.002 | 1.002-1.003 | <0.0001 |  |  |  |  |
| e24UNaE_Kawasaki_ (g/day) | 1.365 | 1.313-1.419 | <0.0001 |  | 1.231 | 1.114-1.360 | <0.0001 |  | 1.230 | 1.114-1.359 | <0.0001 |
| Whole population | | | | | | | | | | | |
| Triglyceride (mg/dL) | 1.003 | 1.003-1.004 | <0.0001 |  | 1.001 | 1.001-1.002 | <0.0001 |  |  |  |  |
| e24UNaE_Kawasaki_ (g/day) | 1.241 | 1.203-1.281 | <0.0001 |  | 1.107 | 1.024-1.197 | 0.0107 |  | 1.115 | 1.027-1.210 | 0.0092 |

*Defined as either the use of antihypertensive therapy and/or systolic BP above 140 mm Hg or a diastolic BP above 90 mm Hg.

Model I, performed using age, sex, and smoking as covariates and body mass index, waist circumference, white blood cell count, hemoglobin, eGFR, fasting plasma glucose, hemoglobin A1c, aspartate aminotransferase, alanine aminotransferase, UACR, and daily alcohol intake as predictors.

Model II, performed using age, sex, and smoking as covariates and body mass index, waist circumference, white blood cell count, hemoglobin, eGFR, fasting plasma glucose, hemoglobin A1c, aspartate aminotransferase, alanine aminotransferase, triglycerides, UACR, and daily alcohol intake as predictors.

OR, odds ratio; *CI*, confidence interval.
