# Supplementary material for: Interactive effect of high sodium intake with increased serum triglycerides on hypertension
Source: PLoS One. 2020 Apr 16;15(4):e0231707. doi: 10.1371/journal.pone.0231707 (PMC7162459; doi:10.1371/journal.pone.0231707)
Supplement: S5 Table — Interactive effect analysis of high sodium intake and increased triglyceride level on hypertension. (DOCX) [file pone.0231707.s007.docx]

S5 Table. Sensitivity analysis. Interactive effect analysis of high sodium intake and increased triglyceride level on hypertension

| Categories | |  | Unadjusted | | |  | Adjusted^#^ | | |
| --- | --- | --- | --- | --- | --- | --- | --- | --- | --- |
| Triglyceride quintile | e24UNaE_Kawasaki_ quintile |  | OR | 95% *CI* | P |  | OR | 95% *CI* | P |
| Participants without antihypertensive medication | | | | | | | | | |
| 1-3 | 1-2 |  | 1 (reference) | |  |  | 1 (reference) | |  |
| 1-3 | 3-5 |  | 1.696 | 1.467-1.962 | <0.0001 |  | 1.233 | 0.881-1.726 | 0.2225 |
| 4-5 | 1-2 |  | 2.749 | 2.371-3.188 | <0.0001 |  | 1.115 | 0.825-1.507 | 0.4767 |
| 4-5 | 3-5 |  | 4.135 | 3.543-4.826 | <0.0001 |  | 1.690 | 1.184-2.413 | 0.0039 |
| Whole population | | | | | | | | | |
| 1-3 | 1-2 |  | 1 (reference) | |  |  | 1 (reference) | |  |
| 1-3 | 3-5 |  | 1.590 | 1.413-1.790 | <0.0001 |  | 1.128 | 0.867-1.467 | 0.3685 |
| 4-5 | 1-2 |  | 2.488 | 2.183-2.835 | <0.0001 |  | 1.314 | 0.972-1.777 | 0.0762 |
| 4-5 | 3-5 |  | 3.331 | 2.976-3.727 | <0.0001 |  | 1.526 | 1.091-2.134 | 0.0137 |

*Adjusted for age, sex, smoking history, body mass index, waist circumference, white blood cell, hemoglobin, eGFR, fasting plasma glucose, hemoglobin A1c, aspartate aminotransferase, alanine aminotransferase, UACR, and daily alcohol intake.
