# Supplementary material for: Interactive effect of high sodium intake with increased serum triglycerides on hypertension
Source: PLoS One. 2020 Apr 16;15(4):e0231707. doi: 10.1371/journal.pone.0231707 (PMC7162459; doi:10.1371/journal.pone.0231707)
Supplement: S6 Table — Index of additive biological interactive effect of high sodium intake and increased triglyceride level on hypertension. (DOCX) [file pone.0231707.s008.docx]

S6 Table. Sensitivity analysis. Index of additive biological interactive effect of high sodium intake and increased triglyceride level on hypertension

|  |  | Unadjusted | | |  | Adjusted* | | |
| --- | --- | --- | --- | --- | --- | --- | --- | --- |
| Measure |  | Estimate | 95% *CI* | P |  | Estimate | 95% *CI* | P |
| Participants without antihypertensive medication | | | | | | | | |
| RERI |  | 0.690 | 0.682-0.697 | |  | 0.342 | 0.335-0.348 | |
| AP |  | 0.167 | 0.054-0.279 | |  | 0.202 | 0.039-0.366 | |
| SI |  | 1.282 | 0.836-1.728 | |  |  |  | |
| Whole population | | | | | | | | |
| RERI |  | 0.051 | 0.045-0.056 | |  | 0.022 | 0.017-0.027 | |
| AP |  | 0.038 | 0.017-0.058 | |  | 0.017 | 0.006-0.028 | |
| SI |  | 1.168 | 1.052-1.284 | |  | 1.010 | 1.007-1.014 | |

*Adjusted for age, sex, smoking history, body mass index, waist circumference, white blood cell, hemoglobin, fasting plasma glucose, hemoglobin A1c, aspartate aminotransferase, alanine aminotransferase, UACR, and daily alcohol intake.

If there was no biological interaction, the 95% *CI* of RERI and AP include 0, and the 95% *CI* of SI contains 1.

RERI, the relative excess risk because of the interaction; AP, the attributable proportion of the interaction; SI, the additive interaction index of synergy
